# Acute Cytomegalovirus Illness in an Immunocompetent Adult Causing Intravascular Hemolysis and Suspected Hemophagocytic Lymphohistiocytosis

**DOI:** 10.1155/2022/7949471

**Published:** 2022-07-08

**Authors:** Ross P. Elliott, Brian P. Freeman, Jeffery L. Meier, Rima El-Herte

**Affiliations:** ^1^Internal Medicine Residency Program, MercyOne Medical Center and Clinics, Des Moines, IA 50314, USA; ^2^Mission Cancer and Blood, Des Moines, IA 50309, USA; ^3^Iowa City Veterans Affairs Health Care System, Iowa City, IA 52246, USA; ^4^Division of Infectious Diseases, Department of Internal Medicine, University of Iowa Carver College of Medicine, Iowa City, IA 52242, USA; ^5^Division of Infectious Diseases, Department of Medicine, Creighton, University School of Medicine and CHI Health, Omaha, NE 68124, USA

## Abstract

**Background:**

Primary cytomegalovirus (CMV) infection of the immunocompetent host usually produces little-to-no illness. Occasionally, the infection results in mononucleosis syndrome, protracted fever, hepatitis, tissue-invasive disease, or Guillain-Barré syndrome. Hemolytic anemia and hemophagocytic lymphohistiocytosis (HLH) are rare complications that have not been reported to co-occur. Having hemolytic anemia in conjunction with more common findings of fever and hepatitis complicates the diagnosis of HLH. *Case Presentation*. A 34-year-old male with previously good health presented with a prolonged febrile illness, jaundice, and anemia. An extensive work-up during hospitalization revealed intravascular hemolytic anemia, leukopenia, hepatosplenomegaly, and biopsy evidence of extensive lymphohistiocytic infiltration of the liver with microgranulomata and sinusoidal hemophagocytosis. Soluble CD25 level was mildly elevated at 1200.3 pg/mL and the HScore calculation (fever, bicytopenia, hepatosplenomegaly, aspartate aminotransaminase 99 IU/L, ferritin 1570 ng/mL, fibrinogen 488 mg/dL, and triglycerides 173 mg/dL) suggested a moderate probability of reactive HLH. Primary CMV infection was diagnosed based on CMV IgM positivity, low CMV IgG avidity index, and low-grade CMV DNAemia. The CMV antigen was not detected in the liver biopsy, and the bone marrow biopsy was unremarkable. The illness began to improve before he received oral valganciclovir for 5 days, and he was in good health 10 months later.

**Conclusion:**

Acute CMV illness in an immunocompetent adult can present with hemolytic anemia and clinicopathologic abnormalities consistent with a form fruste of HLH. The illness is likely due to an excessive or unbalanced immune response that may self-correct.

## 1. Introduction

Human cytomegalovirus (CMV) causes debilitating or fatal diseases in people with immature or profoundly defective cellular immune systems. CMV disease of this severity is rarely encountered in people without these immune system abnormalities. While CMV is the most common cause of the heterophile antibody-negative mono syndrome in adolescents and adults, most CMV infections go unnoticed in immunocompetent people. Occasionally, the first episode of CMV infection in an immunocompetent person causes a self-limited prolonged febrile or atypical monolike illness that is usually accompanied by liver test abnormalities signifying hepatitis [1]. Rarely does the infection progress to a tissue invasive disease that involves the gastrointestinal tract, lung, eye, or other organs [2]. The immunologic response to the acute infection may also at times go awry to produce hemolysis, immune thrombocytopenia, myocarditis, skin eruptions, Guillain-Barré syndrome, meningoencephalitis, and, in rare cases, hemophagocytic lymphohistiocytosis (HLH) [3]. Awareness of the unusual presentations of the acute CMV infection in the immunocompetent host is key to early diagnosis and directing test utilization and disease management.

## 2. Case Presentation

A 34-year-old Caucasian male was in his usual state of good health until July 2020 when he presented with a 1-week history of fever up to 39.3°C, sore throat, headaches, cough, and malaise. Having developed this during the SARS-CoV-2 pandemic, he presented to a COVID-19 testing site where jaundice was noted. He was triaged to the emergency room and then hospitalized. He tested negative for SARS-CoV-2 infection by polymerase chain reaction (PCR). A review of the system was negative for oral ulcers, change in vision, enlarged lymph nodes, chest or abdominal discomfort, dyspnea, nausea, vomiting, diarrhea, genital lesions, dysuria, hematuria, arthralgia, myalgia, neurological change, memory deficit, or rash. He had a cholecystectomy two years ago. He reported no illicit drug use, recent travel, or exposure to sick contacts, tuberculosis, ticks, animals, vaping, herbs, or supplements. He had two young children, both in daycare, and his wife was pregnant. The family medical history was unremarkable; no immunodeficiency, malignancy, autoimmunity, or severe infections. He had used over-the-counter acetaminophen and ibuprofen at the recommended doses. Laboratory tests revealed white blood cell count (WBC) 4.6 k/mm3 (range, 4.5–11 k/mm^3^) with neutrophils 55.6%, lymphocytes 24.2%, and monocytes 13.8%; hemoglobin (Hgb) 13.6 g/dL (range, 13.5–17.5 g/dL); platelets 155 k/mm^3^ (range, 150–450 k/mm^3^); peripheral smear with reactive lymphocytes; alkaline phosphatase 94 IU/L (range, 20–140 IU/L); alanine transaminase (ALT) 219 IU/L (range, 5–55 IU/L); aspartate aminotransaminase (AST) 99 IU/L (range, 5–45 IU/L); total bilirubin 8 mg/dL (range, 0.2–1.5 mg/dL); indirect bilirubin 7.1 mg/dL (range, 0.1–1.4 mg/dL); and normal serum creatinine. Urine analysis showed clear orange urine at pH 6.0 (range, 4.5–7) with specific gravity 1.205 (range, 1.010–1.030), protein 20 mg/dL (negative), ketones 20 mg/dL (negative), urobilinogen >20 (range, 0.1–1.8 mg/dL) and negative values for bilirubin, blood, glucose, nitrite, and leukocyte esterase. Serological testing was negative for hepatitis A virus IgM, hepatitis B virus surface antigen, hepatitis B virus core IgM, hepatitis C virus antibody (Ab), human immunodeficiency virus (HIV) p24 antigen (Ag), HIV-1 and HIV-2 Abs, antinuclear antigen Ab, liver-kidney microsomal Ab, mitochondrial Ab, and antismooth muscle Ab. Ceruloplasmin was normal. Iron level was 219 mcg/dL (range, 35–160 mcg/dL), normal TIBC, elevated iron saturation at 83% (range, 30–50%), and low transferrin at 161 mg/dL (range 200–430 mg/dL). IgG level was normal at 957 mg/dL (range, 650–1600 mg/dL). Blood cultures were negative. Abdominal ultrasound showed moderate hepatomegaly and changes consistent with fatty liver disease. Abdominal MRI showed similar changes plus splenomegaly (16 cm). The patient was discharged home on hospital day 3.

On the following day, he was readmitted because of worsening symptoms and normocytic anemia. The Hgb of 12.5 g/dL on the day of entry steadily decreased to 8.7 g/dL on day 8 of this hospitalization. Other remarkable blood count results included WBC 4.2 k/mm^3^ (range, 4.5–11 k/mm^3^), neutrophils 1.9 k/mm^3^ (range, 1.8–7.7 k/mm^3^), and platelets 155 k/mm^3^. Peripheral smear revealed an increase in reactive lymphocytes but a normal lymphocyte count and no schistocytes. Other remarkable laboratory findings included AST 56 IU/L, ALT 120 IU/L, total bilirubin 7.7 mg/dL, indirect bilirubin 6.7 mg/dL, ferritin 1570 ng/mL (range, 24–336 ng/mL), fibrinogen 488 mg/dL (range, 200–450 mg/dL), triglycerides 173 mg/dL (range, <149 mg/dL), LDH 341 IU/L (range, 135–220 IU/L), haptoglobin 1 mg/dL (range, 40–280), absolute reticulocyte count 0.1947 million/mm^3^ (range, 0.62–1.88 million/mm^3^), plasma free Hgb 60 mg/L (range, 0–9 mg/dL), no urinary hemosiderin, and negative Coomb's test.

**Figure 1:**
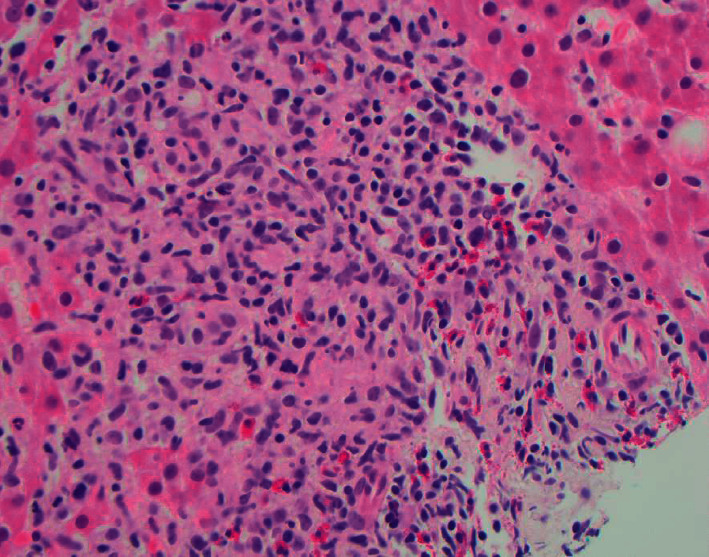
Liver portal triad is expanded by lymphocytes, eosinophils, and noncaseating granulomatous inflammation. H&E stain of liver tissue. No interface hepatitis or proliferating bile ductules. Original magnification, 400x.

**Figure 2:**
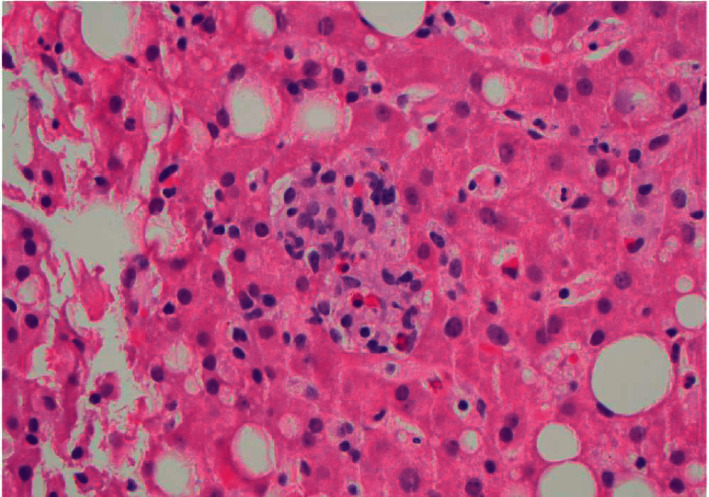
Lobular microgranuloma in the liver. H&E stain of liver tissue. Original magnification, 400x.

**Figure 3:**
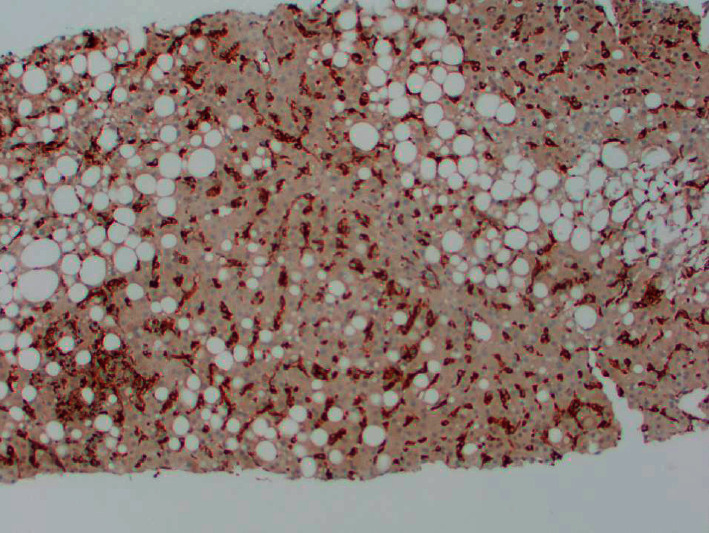
Immunohistochemical staining of CD68-positive macrophages/Kupffer cells (brown stain) in the liver. An excessive number of macrophages are located in sinusoids.

**Figure 4:**
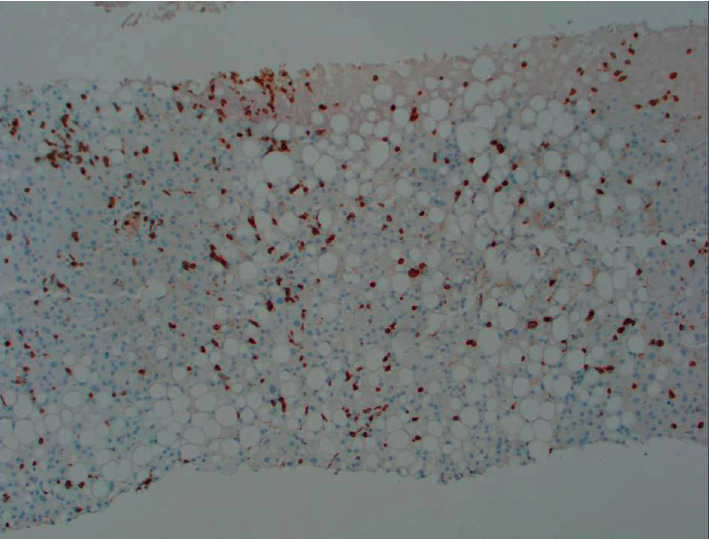
Immunohistochemical staining of CD3-positive T-lymphocytes (brown stain) in the liver. An excessive number of T-lymphocytes are in sinusoids.

**Figure 5:**
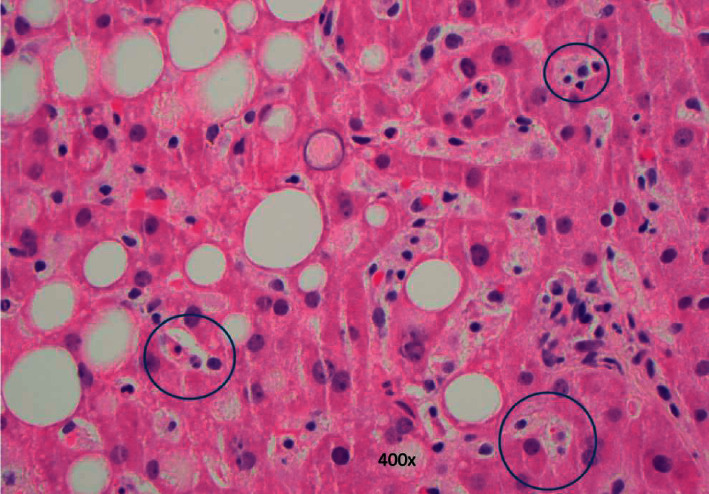
Sinusoidal histiocytic hemophagocytosis. H&E stain of liver tissue. Examples of mature red cells within histiocyte cytoplasm as well as probable degenerate neutrophils within histiocytes are circled. Original magnification, 400x.

Computed tomography of the chest, abdomen, and pelvis showed hepatosplenomegaly without lymphadenopathy. To investigate the cause of the liver abnormalities, a percutaneous liver biopsy was performed on day 2 of hospitalization. This showed portal triad inflammation involving lymphocytes, eosinophils, and granulomatous inflammation (Figure 1), as well as rare lobular microgranulomata (Figure 2). Liver sinusoids had excessive numbers of Kupffer cells (Figure 3) and T-lymphocytes (Figure 4). Occasional benign appearing histiocytes/Kupffer cells had findings of hemophagocytosis of red blood cells and probable leukocytes (Figure 5). Immunohistochemical stains for CMV, HSV-1, and HSV-2 Ags were negative. In situ hybridization for EBV EBER RNA was negative. Special stains for acid-fast bacilli and fungi were negative. Additional testing performed on blood for the HLH possibility included a soluble CD25 (IL-2 receptor) level that was elevated at 1200.3 pg/mL (range, 175.3–858.2). Natural killer cell activity was not assessed.

The infectious disease workup revealed CMV IgM and IgG in serum and CMV DNA in plasma at 41 IU/mL (reference value, undetected; quantification range, 35–10^6^ IU/mL). Primary CMV infection was later confirmed by evidence of a low CMV IgG avidity index of 0.18 (cut off >0.7 for past infection). EBV viral capsid antigen (VCA) IgG and IgM were positive, EBV nuclear antigen IgG >8 (EBNA antibody), and plasma EBV DNA was absent. The blood was negative by PCR for HHV8, ehrlichia, anaplasma, and *Coxiella burnetii* DNA. Also negative in the blood were brucella IgG and IgM, ehrlichia and anaplasma Abs, parvovirus B19 IgM, *Rickettsia rickettsii* Abs, and tuberculosis interferon gamma release assay. Histoplasma Ag in blood and urine; blastomyces Ag in urine, CSF, and blood were negative. Flow cytometry applied to the blood was negative for blasts and atypical lymphoid proliferation. Bone marrow biopsy performed on day 6 of the hospitalization (blood count Hgb 9.8 g/dL), showed normocellular marrow for age with 1+ polychromasia, normal trilineage hematopoiesis, increased storage iron, and no hemophagocytosis, malignancy, CMV Ag, EBV EBER RNA, or stainable acid-fast bacilli and fungi.

## 3. Outcome and Follow-up

The patient's condition was improving before he was given oral valganciclovir for 5 days and discharged home. He received no corticosteroids, immune globulin, or other immunomodulatory therapy. After discharge from the hospital, he had four face-to-face follow-up visits over 10 months. At 5 weeks after the onset of illness, the patient had returned to his usual activity level and had no symptoms of CMV DNAemia. At four months, the cytopenia had resolved (WBC 4.0 k/mm3, Hgb 14.3 g/dL, and platelets 210 k/mm^3^), liver transaminases had normalized, and total bilirubin had improved. At 10 months, the labs showed Hgb 14.5 g/dL, total bilirubin 3.7 mg/dL, direct bilirubin 1 mg/dL, ferritin 280 ng/mL, and haptoglobin 49 mg/dL. The persistent elevation in indirect bilirubin levels could be from residual compensated hemolysis or previously unrecognized Gilbert syndrome. The patient elected to not return for further follow-up.

## 4. Discussion

The extensive workup that was undertaken for our patient revealed hepatosplenomegaly, hepatitis, hemolytic anemia, and neutropenia. Atypical lymphocytes were not present. Testing for many possible etiologies resulted in the diagnosis of primary CMV infection. The diagnosis was based on laboratory findings of low CMV IgG avidity index, CMV IgM positivity, and low-grade CMV DNAemia, in conjunction with a compatible clinical presentation that is discussed below. Serologic evidence of low CMV IgG avidity index or CMV IgG seroconversion confirms a primary CMV infection diagnosis in immunocompetent people, whereas CMV IgM positivity is a product of a primary infection, reactivation/re-infection, or false-positive test [4, 5]. The patient's two young children in daycare may have put him at a higher risk of acquiring CMV, because young children frequently acquire CMV in the daycare setting, shed the virus in high amounts in oral secretions and urine, and transmit CMV to adults via close oral contact with infectious saliva [3, 6]. While acute EBV infection can also produce fever, hepatitis, and hemolytic anemia, our patient's EBV serologies signaled a past EBV infection with immune reactivation (EBV VCA IgM and IgG positivity with highly positive EBNA antibody) or false-positive EBV VCA IgM. Primary CMV infection commonly produces EBV VCA IgM positivity [7, 8]. The lack of EBV DNA in plasma also makes EBV-driven infectious mononucleosis, or HLH, unlikely [9, 10]. Tests for several other possible infectious causes of the patient's illness were negative.

Acute hemolytic anemia is a rare complication of primary CMV infection that was first recognized over 50 years ago [11–13]. Hemolytic anemia arises from intravascular or extravascular hemolysis. CMV may induce antibodies directed against red blood cell surface Ags that may be detected by the direct Coombs test. The majority of case reports of CMV-associated acute hemolysis have indicated the Coombs test was negative and the hemolysis was recognized several days after the onset of any symptoms, as was the situation for our patient. Hepatosplenomegaly is common in these cases and may foster extravascular hemolysis by hepatic and splenic macrophages. Our patient had hepatosplenomegaly with liver biopsy evidence of extensive lymphohistiocytic infiltration and macrophages containing ingested erythrocytes. The self-limited course of acute hemolytic anemia is not unusual [14, 15]. Corticosteroids have been used in severe cases of CMV-induced hemolytic anemia [15, 16], which is in line with the view that corticosteroids may be beneficial in treating the acute hemolytic anemia resulting from EBV-induced infectious mononucleosis [17].

Abnormal liver tests are one of the most common findings in people presenting with symptomatic primary CMV infection [1]. This is usually the consequence of an excessive immunologic response and mononuclear cell infiltration in the liver that may take weeks to resolve. Evidence of active CMV replication is commonly absent in the liver tissue at the time it is examined. The enlarged liver of our patient was marked by a portal and sinusoidal T-lymphocytic and histiocytic infiltration and sinusoidal hemophagocytosis of lymphocytes and RBCs. Microgranulomas were also present. These are features of immune system hyperactivation, including macrophage activation, that are triggered by various types of infections, malignancies, and autoimmune conditions [18]. Histopathologic abnormalities of this type can be associated with or without HLH [18, 19]. HLH is a rare complication of CMV infection in people with acquired or congenital immunodeficiency. Seven cases of CMV-induced HLH have been reported in immunocompetent hosts [20–26], and none of these cases had concomitant hemolytic anemia. These published HLH cases represent the extreme of the illness, and none were reported to be self-limited. The diagnosis of secondary HLH is usually based on criteria provided in the HLH-2004 guideline from the Histocyte Society [27] or in the HScore [28]. The HLH diagnosis per the HLH-2004 guideline requires having 5 of 8 criteria: fever, splenomegaly, cytopenia (2 or 3 lineages), hypertriglyceridemia (triglycerides ≥ 265 mg/dl), hypofibrinogenemia (fibrinogen ≤ 150 mg/dl), hemophagocytosis of bone marrow, spleen, or lymph node, low or absent NK cell activity, elevated ferritin (≥500 ug/L), and elevated soluble CD25 (≥2,400 U/mL) [27]. Our patient met 4 of 8 HLH-2004 guideline criteria for secondary HLH. The HScore differs from the HLH-2004 guideline by including criteria of immunosuppression, hepatomegaly, and elevated AST level and not including NK activity or soluble CD25 level [28]. Our patient did not have immunosuppression or bone marrow hemophagocytosis. His calculated HScore put him at a moderate probability of having reactive HLH. Because EBV-induced infectious mononucleosis and hemolytic anemia elevate ferritin levels [29], an acute CMV monolike illness with hemolytic anemia may do the same. Our patient's CD25 level was not in a range typically observed in HLH cases. Notably, neither the HLH scoring system requires evidence of hemophagocytosis nor lists liver hemophagocytosis as an HLH criterion. However, liver biopsy evidence of lymphohistiocytic infiltration and sinusoidal hemophagocytosis in the setting of an acute systemic inflammatory illness should prompt the consideration of HLH [19, 30].

Our patient's primary CMV infection produced clinical and pathological abnormalities that are consistent with a form fruste of HLH. The illness began to improve before the patient received 5 days of oral valganciclovir and the patient had not received corticosteroid or another hemolysis- or HLH-directed treatment. This suggests that a primary CMV infection presenting in an immunocompetent host as incomplete HLH does not necessarily require HLH-directed therapy. In contrast, the severity of illness and mortality risk from full-blown HLH usually necessitates an HLH-directed intervention, such as corticosteroid, cytotoxic drug, other immunomodulatory agents, and/or hematopoietic stem cell transplantation. Antiviral therapy, such as oral valganciclovir or IV ganciclovir, is often given to inhibit viral replication in the hope of silencing the driver of immune hyperactivation. It was not until HLH was suspected in this patient that CMV infection was considered in the differential diagnosis. Recognizing the varied spectrum of CMV-associated illnesses is key to early diagnosis and directing test utilization and disease management.

## 5. Learning Points

- Acute CMV infection in the immunocompetent host can cause self-limited hemolytic anemia.
- Acute CMV infection can induce lymphohistiocytic infiltration and hemophagocytosis in the liver.
- Intravascular hemolysis complicates the use of current criteria to classify a patient as having HLH.
